# Vibrio albensis bacteremia: A case report and systematic review

**DOI:** 10.1016/j.idcr.2022.e01551

**Published:** 2022-06-30

**Authors:** Ashraf O.E. Ahmed, Gawahir A. Ali, Sara S. Hassen, Wael Goravey

**Affiliations:** a Internal Medicine Department, Hamad Medical Corporation, Doha, Qatar; b Department of Infectious Diseases, Communicable Diseases Center Hamad Medical Corporation, Doha, Qatar

**Keywords:** Vibrio albensis, Non-Vibrio Cholerae, Bacteremia

## Abstract

Vibrio species are gram-negative rods usually known to cause gastroenteritis and infrequently extraintestinal infections in humans. V. cholerae are classically associated with cholera epidemics, particularly serogroup O1 and serogroup O139. However, Vibrio albensis, a non-O1/ non-O-139 serogroup is rarely implicated in human infections. Thus, there is a paucity of data available on the pathogenic profile of V. albensis infections in humans and more research is needed to further delineate the clinical course and management. To fill this gap in the literature, we present the successful management of V. albensis bacteremia in a 64-year-old patient, and we conducted a systematic review of V. albensis infections reported to date, aiming to explore the clinical presentation, course, and management of V. albensis infections.

## Background

Vibrio spp. Infections account for serious illnesses and pandemics. The bacteria are isolated and found in marine and sea animals from where humans contract this pathogen, in addition to the transmission through faecal-oral routes due to poor sanitation and hygiene [1]. More than 100 species of vibrio have been reported; of these, around 12 species are virulent and pathogenic to humans. Vibrio cholera (V. cholera), which produces cholera toxin, is famous among these species, and it was broadly classified as Vibrio cholera (Serogroup O-1 and O-139) and non-vibrio cholera (NOVC, non-O1/ non-O-139 serogroup), which are not producing the cholera toxin [2]. V. cholera presents clinically with severe watery diarrhea as the predominant clinical manifestation, while NOVC has gastrointestinal and extra-gastrointestinal manifestations, commonly skin and soft tissue infections [3]. The diagnosis is confirmed by isolating the organism from the host, and the treatment varies according to the clinical syndrome and antimicrobial susceptibility testing [4].

Vibrio albensis is one of the NOVC species, and similarly, it is waterborne and isolated from sea animals [5]. It is not often reported in the literature to be associated with a specific human disease or a clinical syndrome. In this article, we present a case of V. albensis bacteremia and conduct a systematic literature review of V. albensis infection in humans to study the spectrum of presentation associated with V. albensis and describe the clinical course and the outcomes.

## Case presentation

A 64-year-old woman presented to our emergency department with three days of subjective fever, cough productive of whitish sputum, and shortness of breath on minimal exertion and lying down. She had a prodromal symptom of generalized body ache. She denied chest pain, palpitations, lower limbs edema, or decreased urine output. She had no sick contact. She had a past medical history of endometrial carcinoma for which she underwent chemoradiotherapy, hysterectomy and bilateral salpingo-oophorectomy, and was currently in remission. She also had a history of obstructive uropathy related to endometrial cancer, for which she was on bilateral percutaneous nephrostomy and regular follow up with a urology clinic. She has decompensated liver cirrhosis due to chronic hepatitis B infection for the last 2 years. Her home medication was entecavir. The review of systems was otherwise unremarkable.

On examination, her vital signs were oral temperature of 39.4 C, respiratory rate of 28 breath/min, oxygen saturation of 83% on room air, pulse rate of 130 beats/min, and blood pressure of 130/90 mmHg. On chest examination, she had bilateral breath sounds present, although reduced over the lower-left zone posteriorly and associated with a stony dullness. An abdominal exam revealed a palpable non-tender spleen, and shifting dullness indicating ascites. The rest of the physical examination was non-contributory. Initial investigations showed mild leukocytosis of 12.7 × 10^3/uL (normal 4–10 ×10^3/uL) with neutrophils predominance, and anemia of 8.7 gm/dl (normal 12–15). Her C-reactive protein and procalcitonin were 41 ml/L (normal 0 – 5) and 0.59 (normal < 0.5) respectively. She had normal renal and liver function parameters apart from hypoalbuminemia of 17 gm/L (normal 35 – 50). ECG showed sinus tachycardia, and the chest x-ray showed large left side pleural effusion and consolidation.

The patient was admitted as a case of decompensated liver disease, possibly precipitated by community-acquired pneumonia. She was supplemented with oxygen through a nasal cannula, judicious diuretics, and broad-spectrum antibiotics (meropenem, based on her colonization on multiple admission with E.Coli ESB). A sepsis workup was sent, and therapeutic pleural paracentesis was performed, which showed a transudative effusion. Thereafter, she started to respond clinically with normalization of her vital signs and improvement in the inflammatory markers. Subsequently, the gram`s stained smears revealed the presence of comma-shaped Gram-negative bacilli. The organism was identified as vibrio albensis by the mass spectrometry assisted by flight time desorption/ionization matrix (MALDI TOF-MS, Billerica, MA, USA), with a 99% confidence level.

The antimicrobial susceptibility of this strain was tested based on the CDC recommendation for V. cholerae and an extended battery of other antimicrobials using disk diffusion tests and interpreted according to the Clinical and Laboratory Standards Institute guideline (CLASI) and revealed resistance to ampicillin, ciprofloxacin, tetracycline, ceftriaxone, and trimethoprim/sulfamethoxazole, while sensitive to gentamicin, piperacillin/tazobactam, meropenem and azithromycin.

She was discharged on ertapenem for a total of two weeks after confirming the negativity of repeated blood cultures. Retrospectively, she denied a history of aquatic exposure, raw seafood consumption, or recent travel. On follow-up appointments in the clinic, she was doing well with no evidence of recurrence.

## Methods

### Clinical case reports

We retrospectively search for *vibrio albensis* cases in our institute, Hamad medical corporation, data between May 2009 to March 2022 without age or specimen type limits. Hamad Medical Corporation is the main secondary and tertiary healthcare provider in Qatar. Only this case was reported in Hamad medical corporation during the study period.

### Literature review

We have conducted a systematic literature review in the Medline data through Pubmed and we performed a comprehensive search of Google scholar data without a duration limit. Additional articles were identified by checking the references of relevant articles and duplicates were excluded. We included the articles which described confirmed V. albensis related infections in humans. Our search strategy including the following terms in Pubmed ("Vibrio cholerae non-O1"[MeSH Terms] OR "Vibrio cholerae non-O1"[Text Word] OR "non o 1 vibrio cholerae"[Text Word] OR "non o139 vibrio cholerae"[Text Word] OR "v cholerae non o1 o139"[Text Word] OR "NOVC"[Text Word] OR "vibrio albensis"[Text Word] OR "v. albensis"[Text Word]) AND ("Vibrio Infections"[MeSH Terms] OR “Vibrio Infections*"[Text Word] OR "vibrio bacteremia*"[Text Word] OR "Vibrio cholerae non-O1"[Text Word] OR "vibrio septicemia*"[Text Word]), and ”vibrio albensis infections” in google scholar.

Two authors separately screened the identified articles based on title and abstract. We excluded any Vibrio spp. other than V. albensis, an article written in languages other than English, non-human infections, environmental, and in vitro data. Data were collected including demographics, clinical characteristics, risk factors, microbiological data as well as treatment and clinical outcomes.

### Review results

**Fig. 1:**
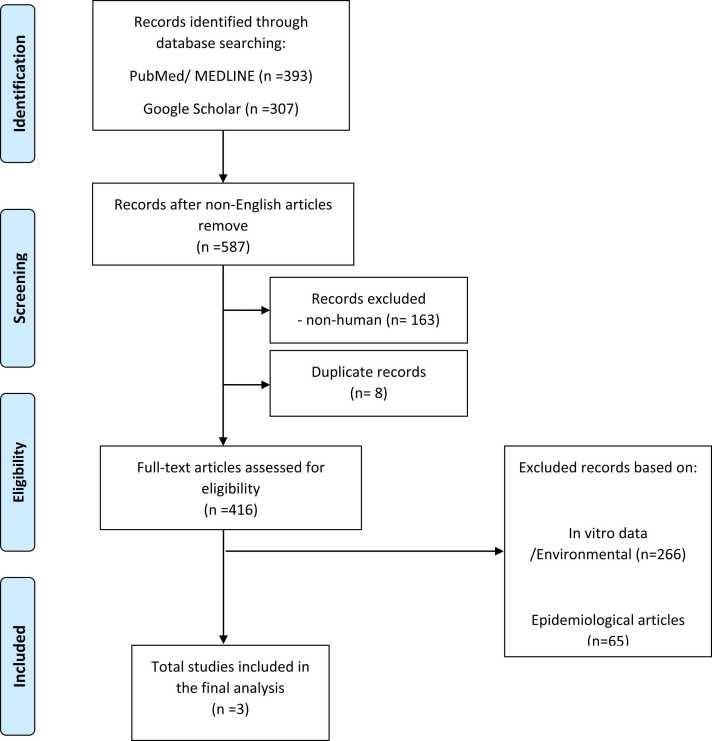
**:** Flow chart from literature search for V. albensis infecitons.

The initial search yielded 393 articles in Pubmed and 307 articles in Google scholar. Eight articles were duplicates (Fig. 1). Of the excluded articles, 113 were non-English articles and 163 were non-human studies. Of the articles screened based on title or abstract, 413 were excluded due to environmental/in vitro data, epidemiological studies, or non-V. albensis infection. We have found only three articles that describe V. albensis related infection (Table 1). The result was congruent between the two reviewers.

**Table 1:** summary of cases described in literature of V. albensis-related infections (including our case reported in this review).

Case number	Age	sex	Possible mode of transmission	Co-morbidities	Presenting symptoms	Diagnoses	Specimen	Antimicrobials used, Duration	Course	Outcome
1. George F Araj[13]	27	Male	Organ isolated from home tap water and a well water (salt water) at home	None	Abdominal pain, nausea and vomiting, dysuria	UTI	Urine	Ciprofloxacin for one week	Uncomplicated	Discharged
2. Madelon F Engel[37]	50	Male	Ready-made tuna ingestion three days prior presentation	COPD, depression, marihuana, and alcohol up use	lethargic and painful discoloration on his right ankle	Septic shock with deep skin infection	Blood culture and Wound (Bullae)	Empirical antibiotics for septic shock (Ciprofloxacin, cefotaxime, polymyxin E, tobramycin, and amphotericin B)	MOF	Died
3. U M Tendolkar[38]	60	Female	None	None	Elective for ICCE	Endophthalmitis	Vitreous fluid	IO injection of chloramphenicol only	Uncomplicated	Decrease visual acuity
Our case	64	Female	None	Liver cirrhosis, History of endometrial malignancy	SOB	Bacteremia and possible pneumonia	Blood	Meropenem for 4 days then Ertapenem, 14 days total	Uncomplicated	Discharged

MOF: multi-organ failure; ICCE: intracapsular cataract extraction; SOB: shortness of breath; IO: Intraocular; UTI: urinary tract infection.

The summary of the screening process is shown in the PRISMA flow diagram (Fig. 1).

## Discussion

Vibrio species are gram-negative, comma-shaped rods isolated mainly from the marine habitat and are pathogenic to humans [6], [7]. V. cholera, V. vulnificus, V. parahaemolyticus, and V. alginolyticus are the most known pathogen in this family [8]. Vibrio cholera is labelled either O group 1(V. cholera O1) or O group 139 (V. cholera O139). This is distinguished by agglutination with serum patients infected by cholera; those species that failed to agglutinate are labelled as non-O1/ non-O-139. cholera [9]. V. albensis is a non-O1/ non-O139 serovar V. Cholerae, and it is a luminescent bacterium that shares more than 70% of the DNA consequences with V. Cholerae [10].

Vibrio cholera causes infections by ingesting the pathogen from undercooked seafood and being exposed to seawater, particularly during the summer and warm climates [11]. The predominant clinical manifestation is gastrointestinal symptoms; severe watery diarrhea, vomiting, and cramping. Patients might also develop life-threatening electrolyte imbalance [12].

V. cholerae non-O1/O139 (NOVC), are not associated with epidemic cholera but can cause a spectrum of illnesses that may range in severity from mild gastroenteritis or UTI to life-threatening septicemia [13]. The pathogenesis of NOVC is less well established; however, the ability to produce Type III Secretion System (TTSS) seems to play a role. This serves to translocate bacterial proteins into eukaryotic host cells and manipulate them during infection [14]. Other virulence factors include hemolysins, RTX toxins and cholix toxin [15], [16]. The latter is associated with self-limited gastroenteritis symptoms [17]. Moreover, few NOVC strains can produce the cholera toxin (CT, encoded by the ctx gene) that has been involved in cholera-like illnesses; however, are less severe than cholera and do not have epidemic potential [18], [19]. Crucially, the rising NOVC-related infections represent one of the most striking examples of emerging human diseases linked to climate change as well the emergence of new pathogenic strains with epidemic potential [20].

Unlike the other Vibrio species, V.albensis can rarely cause infections, and it has not been commonly reported in the literature to be associated with the certain clinical syndrome [13]. Usually, immunocompromised hosts, liver cirrhosis, malignancy and skin wounds are at risk of NOVC bacteremia, however, they rarely cause infections in immunocompetent adults [21]. It is noteworthy that liver cirrhosis is associated with increased prevalence and poor outcomes in NOVC bacteremia; whether this is due to increased comorbidities in cirrhotic patients or unidentified virulence factors still needs to be identified [22]. Moreover, it is postulated that hemolysin expression, increased iron, increased intestinal permeability and abnormal portal vein were the most important factors to explain the susceptibility of patients with liver cirrhosis to NOVC bacteremia [23]. This may explain the situation in our case. We also believe that the occurrence of pneumonia was secondary to the V.albensis bacteremia itself and not the result of a second pathogen causing community-acquired pneumonia. It is important to note that not all patients with NOVC infections have a history of aquatic exposure or raw seafood consumption, as in our patient [7].

The clinical presentations in NOVC infections include gastroenteritis, wound infections, biliary tract infections, pneumonia, meningitis, otitis, urinary tract infections or bacteremia [24], [25].

The reported mortality of NOVC bacteremia is 25–47 % which is slightly lower than that with patients of Cholera caused by V. cholerae (serogroup O1/O139) [26], [27].

The traditional microbiological methods for Vibrio spp identification are time-consuming due to performing a series of biochemical key tests to distinguish closely related species [28], [29]. The Matrix-assisted laser desorption ionization (MALDI) TOF MS demonstrates the ability to distinguish V. albensis from other NOVC because significant interspecies differences exist in clinical relevance, pathogenicity, and antimicrobial susceptibility [30], [31]. Furthermore, PCR based method can be used based on the V. cholerae-specific outer membrane protein gene (ompW) and the O-antigen rfb genes specific for both O1 and O139. NOVC can be confirmed based on the presence of ompW expression and the absence of O1-rfb and O139-rfb genes [32].

There is currently no definitive standard antimicrobials therapy for the treatment of NOVC bacteremia. Thus, antimicrobial-susceptibility testing is critical [33], [34]. However, third-generation cephalosporin, fluoroquinolones, and tetracyclines are suggested first-line agents, followed by macrolides as alternative therapy [23]. However, an increasingly antimicrobial resistance pattern was reported among environmental and clinical NOVC isolates [35]. Therefore, dual-agent therapy with a third-generation cephalosporin and tetracycline or fluoroquinolone was suggested for NOVC bacteremia [36]. Likewise, the optimal treatment for NOVC bacteremia, including V. albensis, is not yet well defined given it is a rarity. Although a median duration of 14 days was suggested by some authors [21] In our case, we treated the patient with a targeted antimicrobial for two weeks given the presence of decompensated liver cirrhosis.

Our systematic review yielded a total of three cases of V. albensis-related infections (Table 1). Cases ranged between 27 and 60 years of age and were predominantly male. Of the identified cases, only one had multiple risk factors and possible chronic liver disease [37]. Of the three cases reviewed, the presenting symptoms are directly related to the organs involved; hence the pathological diagnosis. Interestingly, one out of the three cases was treated by local antibiotics only while various antimicrobials were used to treat the other two [38]. The duration of therapy ranged from 7 to 14 days depending on the clinical syndrome, although data was limited to two cases only. One death was identified in our review though decreased visual acuity was reported in the patient who presented with Endophthalmitis [37].

## Conclusion

Vibrio cholera non-O1/ non-O-139 serogroup bacteremia is a rare clinical entity, and even rarer are V. albensis-related infections that demonstrate the ability of the organism to manifest as an invasive infection. Therefore, careful clinical judgment is needed when encountering NOVC in the blood to differentiate true infection from contamination to avoid devastating consequences especially, in immunocompromised individuals. Treatment should be guided by the pattern of antimicrobial sensitivity and the optimal treatment duration remains unknown, but two weeks is suggested. Furthermore, future research should be directed towards understanding the prevalence, risk factors, and clinical course of V. albensis-related infections, to reduce the related morbidity and mortality rates.

## Funding information

Open access funding provided by Qatar National Library.

## Ethical approval and consent

Written informed consent was obtained from the patient for publication of this case report. A copy of the written consent is available for review by the Editor-in-Chief of this journal on request.

## Author contribution

AA data collection, literature review and manuscript writing and approval; SH data collection, literature review and manuscript writing and approval; GA determine eligibility, data collection, literature review and manuscript writing and approval; WG history and physical, determine eligibility, informed consent, literature review and manuscript writing, manuscript review and approval.

## Conflict of interest

The authors have no conflict of interest to disclose.
